# Biomarkers Affecting Treatment Outcomes of Febrile Neutropenia in Hematological Patients with Lymphomas: Is Presepsin the New Promising Diagnostic and Prognostic Biomarker?

**DOI:** 10.3390/jcm14072238

**Published:** 2025-03-25

**Authors:** Karla Mišura Jakobac, Vibor Milunović, Vesna Kušec, Pero Hrabač, Marko Martinović, Delfa Radić-Krišto, Slobodanka Ostojić Kolonić, Gordana Pavliša

**Affiliations:** 1 Division of Hematology, Department of Internal Medicine, University Hospital Merkur, 10000 Zagreb, Croatia; 2 Department of Innovative Diagnostics, Children’s Hospital Srebrnjak, 10000 Zagreb, Croatia; 3 School of Medicine, University of Zagreb, 10000 Zagreb, Croatia; 4 School of Medicine, Catholic University of Croatia, 10000 Zagreb, Croatia; 5 Department for Respiratory Diseases Jordanovac, University Hospital Centre Zagreb, 10000 Zagreb, Croatia

**Keywords:** febrile neutropenia, presepsin, lymphomas, prognosis

## Abstract

**Background/Objectives**: In hematological patients receiving treatment for lymphomas, febrile neutropenia (FN) is a serious complication associated with significant morbidity and mortality. This prospective study aimed to evaluate the diagnostic and prognostic value of the novel biomarker presepsin (PSP) in episodes of FN in this specific cohort of patients. **Methods:** The study enrolled 37 patients with FN and 18 patients with neutropenia without fever as a control group. Patients with FN were divided into two groups: those with confirmed infections and those without them. Various clinical and laboratory parameters were analyzed, including inflammatory and biochemical markers, focusing on implications of PSP. **Results:** Among patients with FN, 65% had proven infections with significantly higher PSP levels compared to those without infections and control group (*p* < 0.001). Positive blood cultures were found in 13.5% of all FN episodes. PSP showed greater sensitivity than traditional biomarkers like procalcitonin and C-reactive protein for differentiating septic from non-septic complications. Increased PSP levels at admission suggested a poorer survival prognosis. Each 1 ng/mL increase in PSP correlated with a 5% increase in mortality risk (HR 1.05; *p* < 0.001), with a one-year mortality rate of 56.7%, underscoring the necessity for better predictive markers. Other markers, including CRP, PCT, IgG, and albumin, were not significantly associated with mortality; however, platelets and qSOFA exhibited borderline significance. **Conclusions:** PSP is a valuable biomarker for identifying high-risk FN in lymphoma patients and predicting mortality, correlating with infection severity. Larger multi-center studies are needed to validate these findings and optimize PSP’s clinical application to improve outcomes.

## 1. Introduction

In patients with hematological malignancies, such as lymphomas, who are undergoing chemotherapy or immunochemotherapy, FN is a frequent complication that can lead to considerable morbidity and mortality.

Patients with FN should receive their initial doses of empirical antibacterial therapy within one hour of triage. They should be monitored for at least 4 h to assess their suitability for outpatient management, or they should be admitted to the hospital [1,2,3]. Patients with neutropenia are at increased risk of bacterial infections, which are challenging to diagnose due to a lack of local inflammatory responses and clinical signs, resulting in a higher risk of sepsis and septic shock. Early diagnosis of sepsis is critical but difficult, as blood cultures, the gold standard for confirming bacteraemia, have limitations such as lengthy processing times and contamination, leading to false-negative results in up to 40% of patients with clinical signs of sepsis [4,5,6]. Since only 20% of FN cases have microbiologically proven infections, treatment approaches rely on clinical presentation and laboratory evaluations [7]. Current inflammatory biomarkers, C-reactive protein (CRP) and procalcitonin (PCT), are routinely used but have limitations; CRP’s reliability is affected by non-infectious conditions [8,9], while PCT, a reliable marker for bacteraemia, lacks specificity and is not suitable for a standalone use [10,11].

There is a need for improved diagnostic tests or biomarkers for early diagnosis, risk stratification, and prognosis in FN patients [12,13]. Presepsin (PSP), a soluble subtype of CD14 (sCD14-ST), has been investigated as a potential biomarker. CD14 is a glycoprotein involved in innate immunity, existing in membrane-bound (mCD14) and soluble (sCD14) forms. sCD14 is generated from mCD14 or secreted from phagocytes under the influence of cathepsin D [14,15]. mCD14, expressed on macrophages, neutrophils, and dendritic cells, recognizes and binds to bacterial lipopolysaccharides (LPS) [16]. It is then released into circulation as sCD14 and further cleaved into presepsin. PSP may serve as a more sensitive and specific biomarker for sepsis compared to PCT or CRP, with levels increasing rapidly within two hours after infection onset [17]. Elevated PSP levels are associated with Gram-negative infections and can indicate higher levels in non-survivors, suggesting prognostic potential [14,15,16].

FN has significant morbidity and mortality rates ranging from 10% to 30% among hospitalized patients, particularly in those with severe comorbidities [18,19,20]. Mortality rates persist at a high level in the months following episodes of FN, due to the underlying severe immunocompromised status [21]. Despite advances in intensive care, sepsis has a global incidence of about 437 per 100,000 person-years, with approximately 17% in-hospital mortality [22].

Scoring systems like APACHE II, CISNE, qSOFA, SOFA, and MASCC are used to identify high-risk patients, but they are not equally applicable for patients with lymphoproliferative disorders [23,24]. The MASCC index, while rarely used in the hospital setting, is efficient in predicting FN risk based on patient and cancer characteristics. Higher scores indicate lower risk, with a maximum of 26 points. A cutoff value of ≥21 points is used to discriminate patients with low risk from those with high risk (<21 points) for serious FN-related complications [24]. Scoring systems that include biomarkers like CRP and PCT can improve mortality prediction when combined with clinical scores [25,26,27]. PSP is increasingly recognized as a valuable biomarker for predicting mortality in sepsis, with studies indicating that high levels of PSP can improve mortality prediction [28] and may surpass traditional scoring systems in forecasting short-term mortality [29]. Research studies specifically focusing on presepsin in patients with lymphoid malignancies, without including other hematological entities, are limited. Most available studies investigating presepsin within the context of hematological malignancies encompass a broader patient population, including those with various additional disorders. Presepsin has primarily been analyzed in larger cohorts of patients with various heterogeneous comorbidities who were hospitalized for sepsis [30].

This prospective study assessed the diagnostic and prognostic value of PSP in lymphoma patients and FN, investigating its impact on treatment outcomes and patient survival, as well as its association with increased mortality risk at FN diagnosis. We also aimed to identify other reliable predictors of treatment response and survival in FN among immunocompromised individuals.

## 2. Materials and Methods

### 2.1. Study Design and Setting

This prospective single-center study was conducted at the Division of Hematology, Department of Internal Medicine, University Hospital Merkur (UHM), and the Clinical Department of Laboratory Diagnostics of UHM. Part of the analyses was performed at the Department of Innovative Diagnostics, Children’s Hospital Srebrnjak.

The data were collected from March 2020 to April 2023. The study protocol was approved by the hospital ethics committee (approval N° 0311-10481 on 14 September 2019) and was prepared in accordance with the Declaration of Helsinki (1975). Each participant signed an informed written consent form and agreement.

### 2.2. Inclusion Criteria and Definitions

The study subsequently included patients over 18 years of age diagnosed with lymphoproliferative diseases, specifically lymphomas, who developed FN during active chemotherapy or immunochemotherapy treatment. FN is defined by oral temperature of ≥38.3 °C (101.0 °F) or a sustained temperature of ≥38.0 °C (100.4 °F) for one hour, with an absolute neutrophil count (ANC) of <0.5 × 10^9^/L or an ANC expected to decrease to <0.5 × 10^9^/L within 48 h [3].

Patients with FN were grouped based on whether or not there was confirmation of an infection. The confirmation of an infection was defined as the presence of a clearly radiologically documented inflammatory infiltrate, as evidenced by X-ray, multi-slice computed tomography (MSCT), or a microbiologically documented infection confirmed by blood culture, urine culture, or microbiological swab. Patients with a radiologically confirmed infection initially did not have respiratory tract involvement by lymphoproliferative disease and, at the time of FN, showed no signs of progression of the underlying hematological condition. In this group of patients, a microbiological pathogen could not be identified.

Certain patients presented with radiologically confirmed infectious events that were associated with distinctly identified microbiological isolates.

Aside from patients with FN, the study also included additional patients with verified neutropenia without fever who did not meet the criteria for FN diagnosis, constituting the control group. Neutropenia was defined as an absolute neutrophil count (ANC) of less than 1000 per microliter (1000/microL).

### 2.3. Clinical Assessments and Data Collection Procedures

Patients with FN were hospitalized in the Department of Hematology, where they underwent laboratory, microbiological, and radiological evaluations. Following these assessments, empirical antibiotic therapy was initiated and subsequently adjusted as necessary based on the test results.

On the first day of hospital admission in patients with FN (day 1), demographic and clinical data were collected, including age, sex, performance status (ECOG), comorbidities, primary diagnosis, number of lines of therapy, the presence of a central venous catheter (CVC), prophylactic administration of antimicrobial agents, and granulocyte-colony stimulating factor (G-CSF).

Data were also collected from patients in the control group at the time of neutropenia detection, along with blood samples for laboratory analysis (also day 1).

In FN patients, two sets of blood culture samples were collected, along with urine cultures and sputum samples from those with a cough; additional samples were taken as needed based on clinical presentation. A chest X-ray examination was performed on all patients, and if influenza virus infection was suspected, a nasopharyngeal swab was obtained for seasonal flu testing, while PCR testing was conducted for suspected SARS-CoV-2 infection.

All patients (with FN and in the control group) were categorized into three groups:

- Patients with FN and confirmed infection (I group).
- Patients with FN without a confirmed infection (N group).
- Control group representing patients with neutropenic episodes (NEs) without fever (C group).

After blood samples were taken for laboratory tests, appropriate empirical antibiotic therapy was introduced according to the guidelines for the treatment of FN, along with all other necessary supportive treatment measures [2]. The empirical antibiotic therapy included monotherapy with an anti-pseudomonal β-lactam agent such as cefepime, a carbapenem like meropenem or imipenem−cilastatin, or piperacillin−tazobactam. In cases of complications such as hypotension and pneumonia, or when antimicrobial resistance was suspected or confirmed, we added other antimicrobials including aminoglycosides, fluoroquinolones, and vancomycin to the initial regimen.

For each patient with FN, the duration of fever and neutropenia, the length of hospitalization, and the immediate treatment outcomes associated with the current episode were recorded. The severity of FN was assessed by MASCC and qSOFA scoring systems, taking into account the limitations of the qSOFA scoring system specifically in hematological patients [25].

Patients with an MASCC score of 21 points or higher were classified as low-risk for serious complications related to febrile neutropenia, whereas those with an MASCC score below 21 points were considered high-risk. Furthermore, according to the qSOFA scale, patients were also classified into two categories: the lower risk category for the development of sepsis and complications (category 1, which included scores 0 and 1) and the higher risk category (category 2, which included scores 2 and 3).

Patients who survived FN were monitored clinically to evaluate long-term outcomes and overall survival. For all patients, we assessed mortality odds at specified time intervals (i.e., 0–30 days, 31–90 days, 91–180 days, 181–365 days, and beyond 366 days post-FN), to determine the duration of excess mortality associated with FN.

### 2.4. Biomarker Measurements

Laboratory parameters included a complete blood count (CBC), white blood cell count, absolute neutrophil count (ANC), lymphocyte and monocyte count, haemoglobin concentration, platelet count, concentrations of albumin (ALB) and immunoglobulin G (IgG), biochemical indicators of liver and kidney function, and inflammatory biomarkers such as CRP and PCT. In most cases, immune status was also evaluated using flow cytometry. Routine biochemical parameters were obtained from fresh venous blood samples according to standardized procedures in the accredited laboratory of UHM. The detection of PCT was performed using chemiluminescent microparticle immunoassay (CMIA) with an in-house verified linearity of 0.05–100.00 μg/L.

Blood samples for measuring PSP levels were collected on day 1. The blood sample for PSP determination was centrifuged, separated and stored in aliquots at −80 °C until assayed. The PSP concentration was determined using the Human Presepsin ELISA Kit (ref. MBS766136; MyBioSource Inc., San Diego, CA, USA). The manufacturer-claimed linearity was 0.156–10 ng/mL.

### 2.5. Statistical Analysis

Statistical analysis was performed using two software packages: Statistica (Cloud Software Group, Inc., Fort Lauderdale, FL, USA (2023)), Data Science Workbench, version 14, licensed to the School of Medicine, University of Zagreb, and Jamovi (The jamovi project, Sydney, Australia (2024), jamovi (version 2.6) [Computer Software], a kind of open-source software. The level of statistical significance was set at 0.05, with no adjustment for multiplicity.

For variables measured on a nominal or ordinal scale, the results were presented using contingency tables, and group comparisons were performed with the chi-square test. For variables measured on a ratio scale, normality was tested using the Shapiro−Wilk test. Group comparisons were then conducted using appropriate nonparametric methods, specifically the Mann−Whitney U test or Kruskal−Wallis ANOVA.

An expanded analysis of various biomarkers was conducted using a number of univariate Cox analyses to assess their impact on mortality.

Survival analysis was initially conducted using descriptive methods, followed by Kaplan−Meier analysis for each individual predictor. Finally, a survival regression model was constructed using Cox regression.

## 3. Results

The final analysis included 37 patients with FN while the control group comprised 18 patients with NEs without fever. Among the 37 patients with FN, 23 (62.2%) confirmed infectious events, whereas 14 (37.8%) did not.

Within the FN group, there were more women (21; 56.8%) than in the control group (8; 44.4%). This difference in sex distribution was not statistically significant (*p* = 0.391). The mean age across both groups was 62.1 ± 11.9 years (median: 62.0; interquartile range (IQR): 15.5 years), with no statistically significant difference between the groups (*p* = 0.222).

The total of 55 subjects (patients with FN and control group with NEs) were divided into three groups:

- Patients with FN with the proof of infection (“I” group; N = 23);
- Patients with FN without the proof of infection (“N” group; N = 14);
- Patients with NEs without the fever but with the established neutropenia diagnosis (“C” group; N = 18).

The characteristics of the patients and the disease at baseline are summarized in the Table 1.

The most common comorbidity among all patients was arterial hypertension, affecting a total of 12 patients (21.8%), followed by type 2 diabetes mellitus, which was present in 5 patients (9.1%). Hypothyroidism was diagnosed in three patients (5.5%), another three patients (5.5%) had chronic kidney disease, and an additional three patients (5.5%) were diagnosed with hyperlipidemia. Two patients (3.6%) had coronary artery disease, while one patient (1.8%) had hyperthyroidism. Four patients (7.3%) were previously treated for malignant disease, and one patient (1.8%) had post-transplant lymphoproliferative disorder following liver transplantation.

All patients with FN (with or without the proof of infection; groups “I” and “N”; N = 37) actively received treatment for lymphoproliferative disease during the episode of the FN.

Lines of therapy in patients with FN and the control group (patients with NEs) are presented in Table 2.

Within the group of patients with FN and evidence of infection (“I” group; N = 23), 11 patients (47.9%) had only microbiologically documented infections, 7 patients (30.4%) had only a radiologically confirmed sign of infection, and 5 patients (21.7%) met both criteria for confirmed infectious activity.

In two patients (8.7%), *ESBL-producing Klebsiella pneumoniae* was isolated. In four cases of FN (17.4%), multiple pathogenic agents of infection were isolated. In three patients, only microbiological pathogens were isolated, while in one patient, along with multiple microbiological pathogens, a radiological inflammatory infiltrate was also confirmed.

A positive blood culture was confirmed in only five (13.5%) of all patients with FN.

One patient developed a bloodstream infection associated with a CVC, as blood cultures obtained from the patient revealed the same organism isolated from the catheter lumen, which was confirmed to be *Klebsiella pneumoniae*. Additionally, five patients had a positive urine culture, while *Clostridioides difficile* infection (CDI) was confirmed through stool culture in three patients. A microbiological nasopharyngeal swab confirmed the presence of infections in two patients, with one identified as being caused by the SARS-CoV-2 virus and the other by Influenza type A.

Among the Gram-positive bacteria (GPB), *Enterococcus faecalis, Staphylococcus aureus*, and *Streptococcus pneumoniae* were prominent, while the Gram-negative bacteria (GNB) included *Acinetobacter baumannii*, *Escherichia coli*, *Proteus mirabilis*, *Pseudomonas aeruginosa*, *Klebsiella oxytoca*, and *Klebsiella pneumoniae*. Among all patients with proven infection, the source of infection in 12 (52.2%) of them was the respiratory system.

In subjects with proven G- bacterial infection, initial PSP values were statistically significantly (*p* = 0.009) higher (28.5 +/− 20.1 pg/mL) compared to those with no G-bacteria detected (8.93 +/− 11.8 pg/mL).

Surprisingly, no fungal infections were confirmed in any of the patients. However, some radiological infiltrates were described as possibly having a fungal etiology, which we could not confirm through microbiological analysis of the samples. The data are presented in detail in Table 3.

In all patients with FN (i.e., subjects with or without the proof of infection; N = 37; controls excluded), the mean duration of hospitalization was 7.5 days (median = 7; range: 3–16 days), with no statistically significant differences between I and N groups (*p* = 0.959). The mean duration of neutropenia was 4.8 days (median = 4; range: 1–14 days, with no difference between groups; *p* = 0.836). Two patients who died before the third day of illness were excluded from this analysis.

Considering all patients with FN and NEs (i.e., all three groups—N, I, and C), at the first measurement point, PSP levels differed between I (Med; IQR: 7.17 +/− 23.3 ng/mL), N (Med: 3.28; IQR: 4.7 ng/mL), and C (Med: 0.09: IQR: 0.13 ng/mL) groups. The difference between groups was statistically significant (*p* = 0.028) when comparing only the groups I and N, but highly significant differences (*p* < 0.001) were found when comparing all three groups.

**Figure 1:**
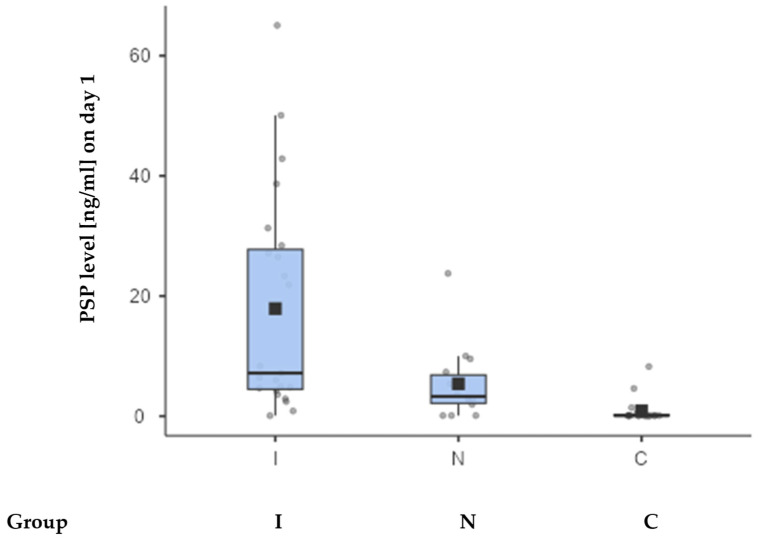
The box-and-whisker plot displaying PSP values between 3 patient groups. The square on the plot represents the mean value, while the horizontal line indicates the median PSP value. Values for each group are in the text, with upper and lower quartiles being 4.47 and 27.8 (group I), 2.15 and 6.85 (group N), and 0.09 and 0.22 (group C).

Figure 1 shows the dispersion in individual measurements between I, N, and C groups.

**Figure 2:**
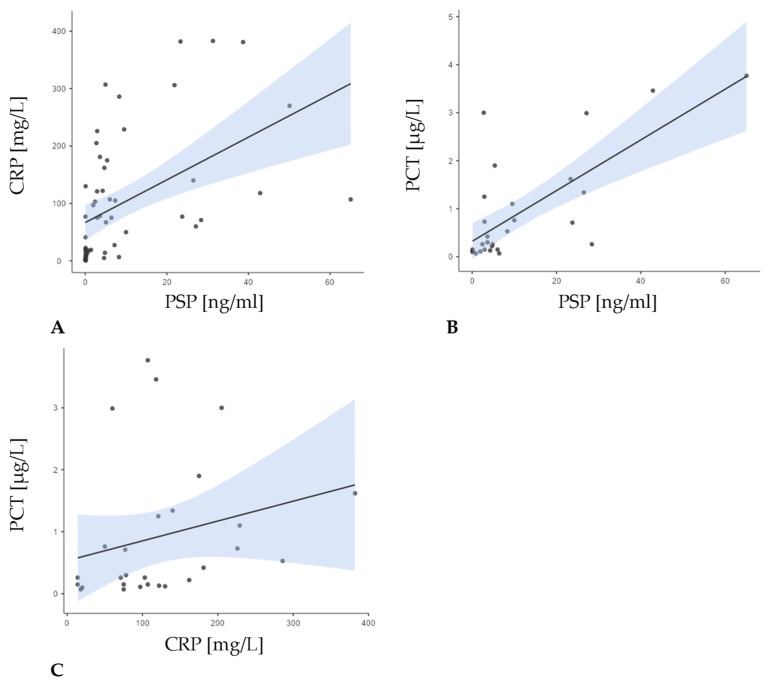
Correlations between PSP levels at baseline measurement and CRP and PCT levels for the entire patient group. A positive correlation was observed between all parameters: (**A**) the correlation between PSP and CRP (r = 0.656; *p* < 0.001); (**B**) the correlation between PSP and PCT (r = 0.601; *p* < 0.001); (**C**) the correlation between CRP and PCT (r = 0.441; *p* = 0.019).

In group I (neutropenia, febrility, and confirmed infection), a strong correlation was found between PSP and PCT (r = 0.638; *p* = 0.008), but not with CRP (r = 0.361; *p* = 0.090). By contrast, in group N (neutropenia and febrility without confirmed infection), PSP correlated with PCT (r = 0.631; *p* = 0.028), and CRP correlated with PCT to some measure (r = 0.564; *p* = 0.056). Finally, among patients in group C (neutropenia only), no significant correlation was observed between PSP and CRP (Figure 2).

**Figure 3:**
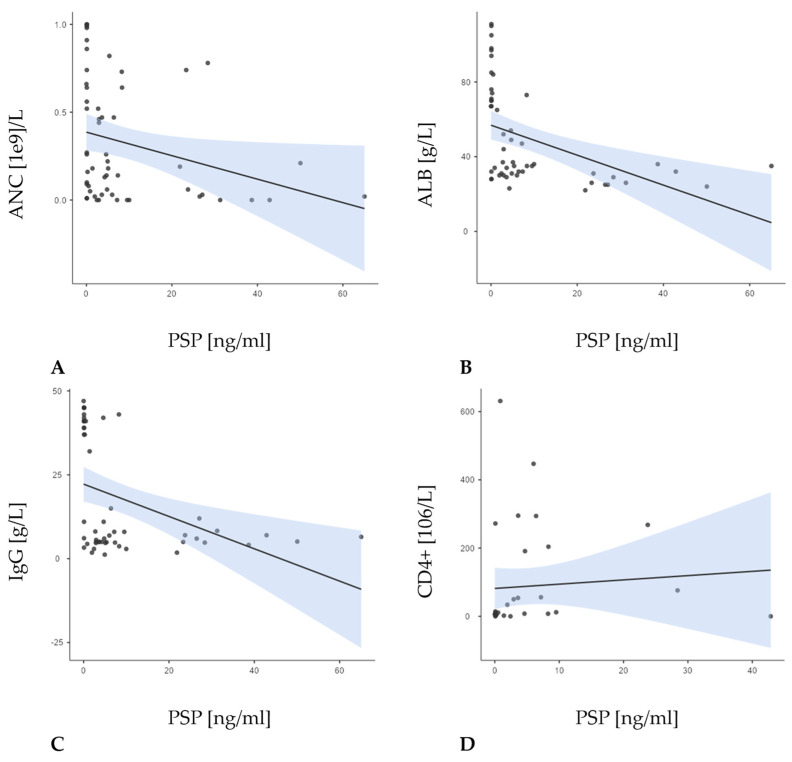
Correlations between the concentration PSP and the number of absolute neutrophil count (ANC) (r = −0.412; *p* = 0.002; (**A**)), concentration of albumin (r = −0.607; *p* <0.001; (**B**)), IgG levels (r = −0.500; *p* = <0.001; (**C**)), and CD4+ levels (r = 0.440; *p* = 0.009; (**D**)) in the overall sample.

In the entire patient sample (N = 55), PSP at baseline demonstrated a significant, moderate negative correlation with both albumin (r = −0.607; *p* < 0.001) and IgG levels (r = –0.500; *p* < 0.001), as well as with the absolute neutrophil count (ANC) (r = −0.412; *p* = 0.002) (Figure 3).

When examining the groups, none of these correlations reached statistical significance, likely owing to smaller sample sizes and differing clinical presentations. In group I, PSP showed generally negative, though non-significant, relationships with ANC, albumin, and CD4+ levels, while its association with IgG was positive. In group N, PSP similarly showed negative trends with ANC and IgG and a positive trend with albumin, though again without reaching significance. Finally, in group C, PSP was negatively correlated with all three parameters, but again not at a significant level.

Other correlations of interest included the correlation between PSP and MASCC (r = −0.188, *p* = 0.266), duration of fever (r = 0.123; *p* = 0.476), and duration of neutropenia (r = 0.037; *p* = 0.834). Furthermore, the duration of fever showed a strong positive correlation to the duration of neutropenia (r = 0.466; *p* = 0.005).

**Figure 4:**
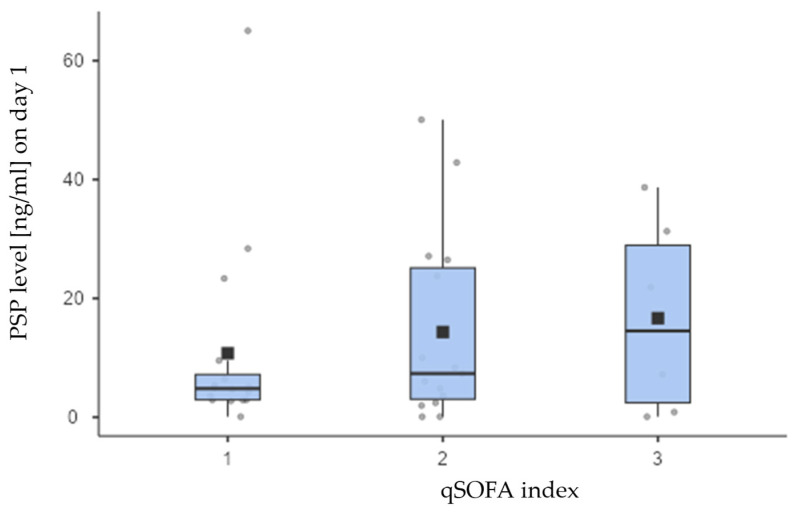
Although both the means (boxes on the chart above) as well as medians (thick lines) of the PSP concentrations differed visibly across the qSOFA scoring parameter groups, these differences were not statistically significant (*p* = 0.733), most likely due to many outlier and extreme measurements notable on the chart. Values for each group are in the text, with upper and lower quartiles being 2.93 and 7.19 (group I), 3.03 and 25.1 (group N), and 2.43 and 28.9 (group C).

Furthermore, the theoretical values of the qSOFA scale range from 0 to 3, and our sample included only categories 1, 2, and 3. Therefore, it is appropriate to assess the statistical significance of differences by dichotomizing the scale into two categories: 1 (which combines the original categories 0 and 1) and 2 (which combines the original categories 2 and 3). However, even with this dichotomization, no statistical significance difference was found between the analyzed groups (*p* = 0.434) (Figure 4).

Additionally, we analyzed survival outcomes for each group and the level of PSP based on the results. The table and graph below illustrate the variations in PSP levels between all three groups of patients. Noticeable are differences in PSP levels between all deceased patients (mean ± SD: 16.0 ± 18.6 ng/mL; median: 5.69 ng/mL) and those who were alive at the time of last visit (mean ± SD: 3.86 ± 6.04 ng/mL; median: 0.51 ng/mL). These differences were statistically significant (*p* < 0.001). Among deceased subjects, there were no statistically significant differences between groups I and N (*p* = 0.126) and between groups N and C (*p* = 0.211), but a significant difference was observed between groups I and C (*p* = 0.029). There were no statistical significance in between-group differences for surviving subjects.

We analyzed survival outcomes in the group of patients with febrile neutropenia based on the evidence of infection, as well as by gender, presence of CVC, and age. The results are presented in figures below.

**Figure 5:**
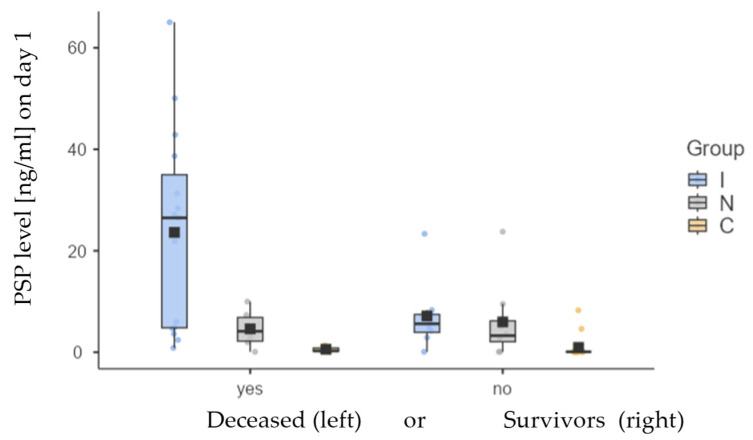
Differences in mean (box) and median (thick horizontal line) values of PSP between deceased and surviving groups for groups I (blue), N (grey), and C (yellow).

**Figure 6:**
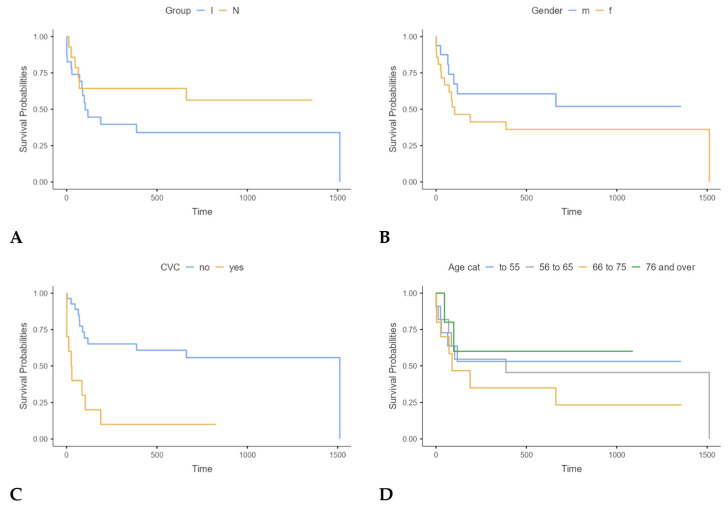
Kaplan−Meier analysis of survival between groups I and N ((**A**); *p* = 0.241); by gender ((**B**); *p* = 0.258); presence of CVC ((**C**); *p* < 0.001); and age ((**D**); *p* = 0.620).

PSP values for deceased and surviving subjects are presented for all three groups (I, N, and C) in Table 4 and Figure 5. Kaplan−Meier analysis (Figure 6), Cox regression, and days survived after sampling (Table 5 below) are shown only for I and N groups since the number of deceased subjects in C group was small (N = 3) and could lead to wrong conclusions.

The results showed that none of the variables had statistically significant impact on mortality, namely CRP (*p* = 0.502), PCT (*p* = 0.280), IgG (*p* = 0.491), albumin (*p* = 0.298), platelets (statistically borderline *p* = 0.090), MASCC index (*p* = 0.103), CD4+ (*p* = 0.981), and qSOFA (*p* = 0.152).

The results indicate that for each increase of 1 ng/mL in PSP concentration, mortality rose by 5% (1.05; 95% CI: 1.02–1.07; *p* < 0.001), which explains 25% of the mortality among our participants (R^2^ = 0.25). Consequently, PSP values are recognized as a major predictor of adverse clinical outcomes.

## 4. Discussion

Our research evaluated the diagnostic and prognostic significance of PSP in patients with lymphoma who experienced FN, emphasizing its influence on treatment outcomes, patient survival, and the correlation with increased mortality risk at the time of FN diagnosis. The study included 37 patients with FN, while the control group comprised 18 patients with neutropenia without fever. Among the patients with FN, 62.2% had confirmed infections (group I), while 37.8% did not (group N). Importantly, our results showed that FN predominantly affected women (56.8%), with a mean age of 62.1 in both groups, with no statistically significant difference between the groups (*p* = 0.222). Furthermore, the sex distribution difference between groups was only borderline significant (*p* = 0.069). These findings underscore the susceptibility of middle-aged individuals undergoing chemotherapy to infections during immunosuppressive treatment. Out of a total of 55 enrolled patients, 52 patients (94.5%) had NHL, and among the episodes with confirmed infections, 91.3% had NHL, emphasizing the relevance of underlying hematological malignancies in FN and their role in predisposing to infectious complications. In addition, it is known that despite the frequent occurrence of neutropenia during the treatment of Hodgkin lymphoma, complications in the form of FN are relatively rare [31].

Our study found that 32.4% of patients with FN had at least one additional health issue, aligning with previous research linking comorbidity burden to increased mortality risk. Future investigations should aim to delineate how specific comorbid conditions interact with PSP levels and other biomarkers to influence FN prognosis. Such insights could pave the way for personalized risk models that incorporate both biomarker data and patient-specific factors [32]. Similar findings have been observed in pediatric patients with oncological and hematological diseases, where FN is a major complication. In these patients, PSP has been evaluated as an early biomarker for bacterial infections, highlighting its potential utility across different age groups. For instance, a study by Arıkan et al. demonstrated that PSP levels were higher in pediatric patients with bacteremia during FN episodes, suggesting its potential as a diagnostic tool in this population. While this investigation focuses on a pediatric cohort of patients with acute leukemia, it has demonstrated that PSP, in conjunction with CRP and PCT, possesses the capability to differentiate between various bacterial infections, notably Gram-negative bacteremia [33]. This finding is corroborated by the results of our own research, which revealed that patients with confirmed Gram-negative bacterial infections exhibited statistically significantly higher initial PSP values (*p* = 0.009).

The documentation of infections within group I revealed that 47.9% of patients had microbiologically confirmed infections, 30.4% had radiologically documented signs of infection, and 21.7% had both. Interestingly, blood cultures were positive in only 13.5% of all cases with FN, highlighting the difficulty of diagnosing bacteremia in those patients. Infections were primarily bacterial, involving Gram-positive pathogens like *Enterococcus faecalis*, *Staphylococcus aureus*, and *Streptococcus pneumoniae* and Gram-negative organisms including *Acinetobacter baumannii*, *Proteus mirabilis, Pseudomonas aeruginosa, Escherichia coli*, and *Klebsiella species*. Viral pathogens, including SARS-CoV-2 and Influenza type A, were also documented, reflecting the broad spectrum of infectious agents complicating FN management. Our findings are consistent with the literature, which indicates that bacteraemia occurs in 10–25% of patients with FN, particularly among those with prolonged or severe neutropenia [2,16].

We demonstrated that PSP levels on day one were significantly elevated in patients with FN and proven infections compared to those without (*p* = 0.028), suggesting its utility as a diagnostic biomarker. PSP levels were highest in group I (infected FN), with a median of 7.17 ng/mL (IQR: 23.3 ng/mL), compared to 3.28 ng/mL (IQR: 4.7 ng/mL) in group N and 0.09 ng/mL (IQR: 0.13 ng/mL) in the control group (group C). The differences between the three groups were statistically significant (*p* < 0.001). PSP showed a moderate positive correlation with CRP (r = 0.656; *p* < 0.001) and a stronger correlation with PCT (r = 0.601; *p* < 0.001), suggesting that PSP tracks closely with PCT, which is highly specific for bacterial infections. However, CRP, which can rise in response to various inflammatory processes, did not correlate as strongly with PSP. These findings also support idea that PSP may be a more bacterial infection-specific biomarker. Compared to other inflammatory markers, such as CRP and PCT, it has the ability to predict the severity of a bacterial infection, and the measurement can be performed using a simple procedure that takes less than 17 min, as previously highlighted in the work of Memara et al. [16,34].

In group I, PSP correlated strongly with PCT (r = 0.638; *p* < 0.008), but not with CRP, supporting its role as an infection-specific marker. In contrast, in group N (febrile neutropenia without proven infection), neither PSP nor PCT showed significant correlations, indicating that elevated PSP levels are more indicative of bacterial infection than general inflammation [34]. In group C, consisting of patients with neutropenia only, no significant correlation between PSP and CRP was found, with PCT data unavailable. This lack of association likely reflects the absence of any established infectious or inflammatory process in this cohort. PSP also showed significant negative correlations with albumin (r = −0.607; *p* < 0.001) and IgG levels (r = −0.500; *p* < 0.001), as well as a weaker but significant inverse relationship with the absolute neutrophil count (ANC) (r = −0.412; *p* = 0.002). These findings suggest that higher PSP levels may reflect both the severity of infection and the depletion of immune or nutritional reserves [16,32]. PSP show a significant correlation with CD4+ levels (r = 0.440; *p* = 0.009). These group patterns suggest that while PSP may broadly track with certain laboratory parameters in a larger, more heterogeneous population, the strength and direction of these relationships can shift, depending on the underlying clinical context, particularly the presence or absence of active infection.

The results of our study may indicate that PSP is a good prognostic marker in patients with lymphoma and FN, and increased PSP values are associated with poorer treatment outcomes and increased mortality. Patients with FN and low MASCC scores had increased PSP concentrations, reinforcing its role as a prognostic marker for severe FN complications. However, no correlation was found between PSP and qSOFA scores. Other studies show a weak positive, negligible, or insignificant correlation between PCT, CRP, and PSP with the SOFA score, for example, the study by Moustafa et al., which analyzed 60 patients with acute leukemia and FN [35]. Several studies indicate that PSP serves as a highly sensitive and specific biomarker for sepsis, with its concentration exhibiting a strong correlation with the severity of the sepsis syndrome and in-hospital mortality [36,37]. Notably, marked elevations in PSP levels have been associated with adverse clinical outcomes, thereby reinforcing its utility in prognostic evaluations. A study published in *the Journal of Leukocyte Biology* demonstrated that increased levels of PSP over several days were linked to higher mortality rates among patients in the intensive care unit [38]. Similar to other infection biomarkers, serial PSP measurements can assist in prognostic stratification of septic patients, where elevated levels are linked to higher mortality and greater risk of severe complications [39].

Patients with FN and elevated PSP levels experienced prolonged neutropenia and extended hospital stays, echoing findings in sepsis studies where PSP levels correlated with disease severity and in-hospital mortality [39,40,41]. Mortality rates were high in our study, with 56.7% of patients dying within one year. Among those who died, PSP levels were significantly higher than in survivors, with a mean of 16.0 ng/mL (SD 18.6) compared to 3.86 ng/mL (SD 6.04) in survivors (*p* < 0.001). Cox regression analysis demonstrated that each 1 ng/mL increase in PSP was associated with a 5% increase in mortality risk (HR: 1.05; 95% CI: 1.02–1.07; *p* < 0.001), explaining 25% of the observed mortality variance (R^2^ = 0.25). We consider a noteworthy aspect of our study to be that it was conducted in a homogeneous group of patients with the lymphoproliferative disease, dominant NHL. Top of Form

Previous extensive meta-analyses like the study by Molano-Franco et al., which included 15,681 patients, have not demonstrated a connection between the basal and isolated measurement of PSP and mortality prognosis among critically ill septic patients during their hospital stay likely due to the heterogeneity of the patients and their underlying conditions [42]. On the other hand, emerging evidence suggests the prognostic significance of PSP as a predictor of mortality, derived from studies involving a smaller cohort of septic patients in an intensive care unit (ICU), where PSP levels were assessed during the early phases of bacteremia. The key findings of the study by Abdelshafey et al. confirm that the measurement of PSP is superior in the early identification of sepsis and in predicting mortality compared to traditional methods such as SIRS and qSOFA. However, the main limitation of the study is the small sample size, which may restrict the generalizability of the results to a broader patient population, along with the incomplete assessment of potential comorbidities that may impact the outcomes [43].

Investigating the impact of PSP levels measured on the third day of illness on mortality in our cohort of patients would be valuable, especially considering that the progressive dynamics of PSP may indicate an inadequate response to treatment for FN. Research has shown that the dynamics of PSP levels influence mortality in immunocompromised patients with sepsis. However, most studies investigating the impact of PSP on mortality were conducted in ICUs, where patients with sepsis unrelated to their underlying conditions were treated, unlike in our studied patient cohort.

While PSP was a significant prognostic factor, other commonly used biomarkers, including CRP, PCT, IgG, albumin, and MASCC scores, did not show statistically significant associations with mortality (*p* > 0.05). Platelets and the qSOFA index showed borderline significance (*p* = 0.090 and *p* = 0.152, respectively), further suggesting that conventional risk-scoring methods may be inadequate for FN patients and that dynamic biomarkers like PSP offer superior predictive insights. Previous studies have demonstrated similar findings regarding the prognostic value of PSP. PSP has consistently shown to be a superior prognostic factor compared to CRP, although it lacks significant advantages over PCT and other biomarkers [40,41,42,43,44,45].

Beyond its prognostic value, PSP offers potential for monitoring therapeutic responses in FN. Serial measurements of PSP may provide insights into the efficacy of ongoing treatments, allowing clinicians to adjust interventions in real time [46]. For example, declining PSP levels during treatment could indicate effective infection control, while persistently high levels may signal the need for alternative therapeutic strategies or intensified care. This dynamic approach could significantly enhance the precision of FN management, particularly in high-risk groups with complex medical histories. Moreover, PSP’s role in perioperative medicine highlights its potential in monitoring infection risk in various clinical settings [47].

Our study had several limitations, primarily its small sample size and the impact of the COVID-19 pandemic, which led to prophylactic G-CSF use in many patients to prevent treatment-related complications. In addition to the markers we investigated in our study, there are certainly other patient and disease parameters that influence the outcomes of FN treatment, as well as overall mortality, and further research is needed [20]. Despite these limitations, our findings clearly demonstrate that PSP is a significant diagnostic and prognostic biomarker in FN patients with lymphoproliferative diseases, highlighting the need for further investigations and evidence.

## 5. Conclusions

FN represents a critical and potentially life-threatening complication in individuals receiving treatment for lymphoproliferative disorders, particularly NHL. Despite advancements in diagnostic methodologies and therapeutic strategies, the mortality rates associated with FN remain persistently elevated.

Our study demonstrated that patients with lymphomas, predominantly NHL, who had increased PSP at admission, had poor survival prognosis. Elevated PSP levels on the first day of illness correlate strongly with infection severity and mortality, identifying high-risk patients with febrile neutropenia and underscoring its potential role in guiding clinical decision-making.

However, further larger multi-center studies are required to validate our findings and establish the most effective clinical applications of presepsin in managing FN. By integrating PSP measurement into routine practice, clinicians may improve risk stratification and tailor treatment strategies to enhance patient outcomes in this vulnerable population.

## Figures and Tables

**Table 1:** Characteristics of all patients and the disease.

	Patients with FN and Proven Infection (Group I) (n = 23; 41.8%)	Patients with FN Without Proven Infection (Group N) (n = 14; 25.5%)	Patients with NEs (Group C) (n = 18; 32.7%)	Total (n = 55; 100.0%)	*p*-Value
Male Female	8 (34.8%) 15 (65.2%)	8 (57.1%) 6 (42.9%)	10 (55.6%) 8 (44.4%)	26 (47.3%) 29 (52.7%)	0.289
ECOG 0 ECOG ≥ 1	8 (34.8%) 15 (65.2%)	10 (71.4%) 4 (28.6%)	12 (66.7%) 6 (33.3%)	30 (54.5%) 25 (45.5%)	0.043
IgG < 4 g/L	5 (21.7%)	2 (14.3%)	1 (5.6%)	8 (14.5%)	0.345
CVC					0.139
Yes	8 (34.8%)	2 (14.3%)	2 (11.1%)	12 (21.8%)	
no	15 (65.2%)	12 (85.7%)	16 (88.9%)	43 (78.2%)	
MASCC score ≥ 21	1 (4.3%)	3 (21.4%)	0 (0.0%)	4 (10.8%)	-
qSOFA ≥ 2	14 (60.9%)	7 (50%)	0 (0.0%)	21 (56.8%)	-
Use of G-CSF following the protocol					0.127
yes	17 (73.9%)	7 (50.0%)	8 (44.4%)	32 (58.2%)	
no	6 (26.1%)	7 (50.0%)	10 (55.6%)	23 (41.8%)	
Use of antimicrobial prophylaxis					0.786
acyclovir	22 (95.7%)	11 (78.6%)	18 (100.0%)	51 (92.7%)	
co-trimoxazole	12 (52.2%)	6 (42.9%)	14 (77.8%)	32 (58.2%)	
fluconazole	11 (47.8%)	4 (28.6%)	5 (27.8%)	20 (36.4%)	
ciprofloxacin	4 (17.4%)	1 (7.1%)	0 (0.0%)	5 (9.1%)	
Subtype of lymphoproliferative disease:					0.819
non-Hodgkin lymphoma	21 (91.3%)	13 (92.9%)	18 (100.0%)	52 (94.5%)	
Hodgkin lymphoma	2 (8.7%)	1 (7.1%)	0 (0.0%)	3 (5.5%)	
Line of therapy					0.109
1st	14 (60.9%)	5 (35.7%)	13 (72.2%)	32 (58.2%)	
≥2nd	9 (39.1%)	9 (64.3%)	5 (27.8%)	23 (41.8%)	
No comorbidities	13 (56.5%)	12 (85.7%)	4 (22.2%)	29 (52.7%)	0.178

FN—febrile neutropenia, NEs—neutropenic episodes; ECOG—Eastern Cooperative Oncology Group; IgG—Immunoglobulin G; CVC—Central venous catheter; MASCC—Multinational Association for Supportive Care in Cancer; qSOFA—quick Sepsis-Related Organ Failure Assessment; G-CSF—Granulocyte colony-stimulating factor.

**Table 2:** Number of patients with FN and NEs according to treatment lines.

Line of Therapy	Patients with FN N (%)	Patients with Nes N (%)
1	19 (51.4)	13 (72.2)
2	5 (13.5)	3 (16.7)
3	6 (16.2)	2 (11.1)
4	2 (5.4)	0 (0.0)
5	5 (13.5)	0 (0.0)

FN—febrile neutropenia; NEs—neutropenic episodes.

**Table 3:** Characteristics of FN with proven infection (the “I” group; N = 23), including the type of pathogen or site of infection.

FN with Proven Infection	(Group I, N = 23)
Only microbiologically documented infection	**(N = 11; 47.9%)**
GNB	4 (17.4%)
- *Acinetobacter baumannii*	1 (4.3%)
- *Escherichia coli*	1 (4.3%)
- *Proteus mirabilis*	1 (4.3%)
- *Klebsiella pneumoniae ESBL*	1 (4.3%)
GPB	2 (8.7%)
- *Enterococcus faecalis*	1 (4.3%)
- *Staphylococcus aureus*	1 (4.3%)
Viral	2 (8.7%)
- SARS-CoV-2 virus	1 (4.3%)
- Influenza A virus	1 (4.3%)
Polymicrobial	3 (13.0%)
- *Klebsiella pneumoniae ESBL*, *Escherichia coli* and *Enterococcus faecalis*	1 (4.3%)
- *Klebsiella pneumoniae* and *Pseudomonas aeruginosa*	2 (8.7%)
Only radiologically documented infection	**(N = 7; 30.4%)**
Pneumonia	6 (26.1%)
Sinusitis	1 (4.3%)
Microbiologically and radiologically documented infection	**(N = 5; 21.7%)**
*Klebsiella pneumoniae*, CDI, *Enterococcus faecalis* and pneumonia	1 (4.3%)
CDI and pneumonia	2 (8.7%)
*Streptococcus pneumoniae* and sinusitis	2 (8.7%)

CDI—Clostridioides difficile infection; ESBL—extended-spectrum beta-lactamase; FN—febrile neutropenia; GNB—Gram-negative bacteria; GPB—Gram positive bacteria.

**Table 4:** Differences in PSP levels in deceased and surviving patients in all three studied groups.

PSP Levels [ng/mL] on Day 1							
Mortality Outcome	Group	N	Mean	Median	SD	Minimum	Maximum
yes	I	15	23.622	26.4900	19.824	0.8500	65.04
	N	6	4.612	4.1450	3.670	0.0900	9.99
	C	3	0.587	0.2600	0.718	0.0900	1.41
no	I	8	7.164	5.6150	7.029	0.0900	23.34
	N	8	5.981	3.2800	7.785	0.0900	23.76
	C	15	0.959	0.0900	2.333	0.0000	8.27

**Table 5:** Deceased subjects by survival time, categorised.

Mortality Days	N	%
up to 30	8	21.6%
31–90	8	21.6%
91–180	3	8.1%
181–365	2	5.4%
366 and more	16	43.2%

## Data Availability

No new data were created or analyzed in this study. Data sharing is not applicable to this article.

## Abbreviations

ANC	Absolute neutrophil count
ALB	Albumin
APACHE II	Acute physiology and chronic health evaluation II
CBC	Complete blood count
CDI	Clostridioides difficile infection
CISNE	Clinical index of stable febrile neutropenia
CMIA	Chemiluminescent microparticle immunoassay
CVC	Central venous catheter
CRP	C-reactive protein
ECOG	Eastern Cooperative Oncology Group
ELISA	Enzyme-linked immunosorbent assay
FN	Febrile neutropenia
G-CSF	Granulocyte colony-stimulating factor
GNB	Gram-negative bacteria
GPB	Gram-positive bacteria
ICU	Intensive care unit
IgG	Immunoglobulin G
IQR	Interquartile range
MASCC	Multinational Association for Supportive Care in Cancer
MED	Median
mCD14	Membrane-bound form of CD14
NE	Neutropenic episode
NHL	Non-Hodgkin lymphoma
PCR	Polymerase chain reaction
PCT	Procalcitonin
PSP	Presepsin
qSOFA	Quick Sepsis-related organ failure assessment
SD	Standard deviation
sCD14-ST	Soluble subtype of CD14
SOFA	Sequential organ failure assessment
UHM	University Hospital Merkur
WHO	World Health Organization
